# Is there an association between spatial access to parks/green space and childhood overweight/obesity in Calgary, Canada?

**DOI:** 10.1186/1479-5868-6-77

**Published:** 2009-11-20

**Authors:** Melissa L Potestio, Alka B Patel, Christopher D Powell, Deborah A McNeil, R Daniel Jacobson, Lindsay McLaren

**Affiliations:** 1 University of Calgary, Department of Community Health Sciences, Faculty of Medicine, 3330 Hospital Drive NW Calgary, Alberta, T2N 4N1, Canada; 2 University of Calgary, Bachelor of Health Sciences program, (at time of writing) Faculty of Medicine, 3330 Hospital Drive NW Calgary, Alberta, T2N 4N1, Canada; 3 University of Calgary, Faculty of Nursing and Population and Public Health, Alberta Health Services, 2888 Shaganappi Trail NW Calgary, Alberta, T3B 6A8, Canada; 4 University of Calgary, Department of Geography, Faculty of Social Sciences, 2500 University Drive NW, Calgary, AB, T2N 1N4, Canada

## Abstract

**Background:**

The recent increase in childhood obesity is expected to add significantly to the prevalence of chronic diseases. We used multivariate multilevel analysis to examine associations between parks/green space and childhood overweight/obesity across communities in Calgary, Canada, a city characterized by intensified urban sprawl and high car use.

**Methods:**

Body Mass Index was calculated from measured height and weight data obtained from 6,772 children (mean age = 4.95 years) attending public health clinics for pre-school vaccinations. Each child's home postal code was geocoded using ESRI ArcGIS 9.2. We examined four measures of spatial access to parks/green space (based on Geographic Information Systems): 1) the number of parks/green spaces per 10,000 residents, 2) the area of parks/green space as a proportion of the total area within a community, 3) average distance to a park/green space, and 4) the proportion of parks/green space service area as a proportion of the total area within a community. Analyses were adjusted for dissemination area median family income (as a proxy for an individual child's family income) community-level education, and community-level proportion of visible minorities.

**Results:**

In general, parks/green space at the community level was not associated with overweight/obesity in Calgary, with the exception of a marginally significant effect whereby a moderate number of parks/green spaces per 10,000 residents was associated with lower odds of overweight/obesity. This effect was non-significant in adjusted analyses.

**Conclusion:**

Our null findings may reflect the popularity of car travel in Calgary, Canada and suggest that the role built environment characteristics play in explaining health outcomes may differ depending on the type of urban environment being studied.

## Background

Recently the public health literature has seen an increasing number of studies investigating the relationship between various attributes of place and the health of populations [1-5]. This type of research strives to explain geographic variations in health outcomes as a function of both place and the individual characteristics of people living in that place [6,7]. As one example, there has been growing interest in understanding the role of residential environments in enhancing and constraining physical activity and influencing obesity[8]. This is particularly relevant considering evidence of a notable increase in the prevalence of obesity in Canadian children over recent decades[9,10]. Increasing prevalence of childhood obesity raises concern because childhood obesity has implications for health during childhood and into adulthood[11,12]. Increased childhood BMI, which often tracks into adulthood, has an important influence on adult morbidity, notably for cardiovascular disease, diabetes, and some cancers[12].

The increasing prevalence of pediatric obesity has drawn attention to children's decreasing physical activity levels[13]. Research has shown that physical activity levels established in youth tend to track into adulthood, and physical activity promotion in youth may facilitate a carryover of healthful habits into adulthood[14]. Promotion of physical activity is therefore a priority in current public health policies. However, to promote physical activity among children, its determinants need to be understood. From an ecological perspective, behaviours are not only affected by personal characteristics (e.g., age) but by interactions with the larger social and cultural contexts in which children live [15-17]. Research to test assertions of this perspective have examined how aspects of the built environment put some children at increased risk for obesity by encouraging the consumption of energy-dense foods and discouraging physical activity[15,17-19].

Disparities in chronic disease risk factors, such as obesity, may be partially attributed to neighbourhood environments that are poor in resources that could support healthy behaviours[20]. Neighbourhoods may have an impact on obesity and related health behaviours of young children in particular, as they spend much time in these environments[21]. For example, Liu and colleagues (2007) concluded that after controlling for individual socio-demographic variables and neighbourhood socioeconomic status, measures of decreased vegetation significantly predicted overweight in children [22]. A Canadian study explored whether aspects of neighbourhood design and spatial access to physical activity facilities were associated with body weight status among young children and found that increased walkability of neighbourhoods and intersection density were associated with lower odds of being overweight/obese among preschool girls but not boys[23]. Another Canadian study found that after controlling for individual/family factors, children living in the 'lowest-income' (i.e., largest proportion of people living below the low income cut-off) neighbourhood had an increasing BMI percentile over time compared to those children living in a 'middle-income' neighbourhood[24]. However, the mechanisms by which this occurred are unclear.

Ferreira and colleagues (2006) conducted a systematic review on environmental correlates of youth physical activity[25]. Among these studies, features of the built environment such as access to equipment, facilities, or programmes were investigated most often, but were generally unrelated to physical activity. However, other studies that measured spatial access to recreational facilities, including parks, have shown that access is associated with increased physical activity in youth [26-28]. Some studies that examine the association between spatial access, measured either objectively or subjectively, to various types of recreational space (e.g., indoor facilities, outdoor parks) and childhood overweight/obesity status, show significant inverse associations [26,27]. For example, Veugelers and colleagues (2008) found that grade five children in Nova Scotia, Canada, who lived in neighbourhoods where parents perceive good (as opposed to poor) spatial access to playgrounds and parks, were less likely to be overweight or obese[27]. Gordon-Larsen and colleagues (2006) objectively measured community-level spatial access to a variety of types of recreational facilities/parks and found that children with access to more facilities/parks were less likely to be overweight[26]. However, another study conducted with low-income pre-school children in the US showed no association between objectively-measured proximity to playgrounds and childhood obesity[28]. A Canadian study examined the number of physical activity facilities (including parks and playgrounds) in a child's neighbourhood and found no association with body weight status[23]. However, the authors note that playground distribution is very equitable in the city studied, which may explain the lack of associations. These studies suggest that spatial access to parks/green space may be an aspect of the built environment that is associated with childhood overweight/obesity.

Limitations of earlier studies on the relationship between obesity and the built environment in children include a reliance on self or parent reported data for BMI, a lack of objectively measured data for physical features of the environment, and limited discussion of how associations may be context-specific; for example, presence and proximity of parks may be important in areas with high pedestrian activity but less important in car-dominated areas. The study undertaken addresses these limitations by using measured height and weight data for a large representative sample of children and Geographic Information Systems (GIS) to objectively measure four dimensions of spatial access to parks/green space. GIS has proven valuable when studying spatial access to parks [29,30] and elements of the built environment that relate to obesity[31].

The aim of this study is to examine the association between spatial access to parks/green space, measured in four different ways, and childhood overweight/obesity in the specific context of Calgary, Canada. We focus our analysis on spatial access to parks/green space by including measures of ease with which parks/green space can be reached (e.g., distance) as well as the proportional area or number of parks/green space within communities. Calgary is one of Canada's major metropolitan areas and is characterized by rapid population growth, which has led to intensified urban sprawl. According to recent data from Statistics Canada, Calgary is a city characterized by more car travel than nearly all other major Canadian centres [32,33] and thus provides a unique opportunity to examine these associations and explore the conditions under which specific built environment features may or may not matter.

## Methods

### Data Source and Participants

Measured height and weight data were obtained from 8,401 children in Calgary, Canada who presented to a public health clinic for their regular (scheduled to occur before entering grade one) vaccination between January 2005 and January 2006. Region statistics indicate that 80% of children are vaccinated at these clinics and therefore the majority of children were captured[34]. The detailed data collection protocol has been described elsewhere[34]. We used each child's measured height and weight to calculate their BMI and classify each child as: neither overweight nor obese, overweight, or obese according to the international BMI cut-off points established by Cole and colleagues (2000)[35]. These international cut-offs are recommended for research purposes in Canada[36]. As part of the data collection process, a home postal code was recorded. Each record was geocoded by postal code using the Postal Code Conversion File within ESRI ArcGIS 9.2[37]. Ethics approval was obtained from the University of Calgary's Conjoint Health Research Ethics Board.

### Geographic Unit of Analysis

In Calgary, there were 185 distinct communities as of 2001. Communities in Calgary are reasonable territorial units for the study of small-area exposures because they were constructed on the basis of social, historical and geographic criteria[5]. Calgary communities are locally relevant and well-known to residents[5].

### Measures

Age and sex were recorded when the child visited the public health clinic. No other individual-level sociodemographic information was available. As a proxy for an individual child's family income we obtained data on the median dissemination area (DA) family level income from the 2001 Canadian census (DAs are the smallest geographic unit at which the census is publicly distributed. They vary in size but are created to contain a uniform population (400 to 700 people) within its boundaries)[38]. Also from the census, we obtained data on community-level education (proportion of adults in a community with at least a completed Bachelor's degree), and the proportion of the total community population that is a visible minority.

Parks/green space in this study refers to all public parks, schools (i.e., school fields) and recreation areas (e.g., public riverfront) within the city (data retrieved from the City of Calgary [39]). Parks/green space that were greater than one km^2 ^(e.g., provincial parks) were deemed to serve the entire population of the city [40] and were removed from this analysis. Our first measure of access to parks/green space was a simple count of the number of parks/green space (based on their area centroid) per 10,000 residents (based on the 2001 population). Our second measure represented the geographic area (km^2^) of parks/green spaces as a proportion of the total area within a community. To account for the likelihood that people cross community boundaries to visit nearby parks/green space we used two additional measures of access to parks/green space applying current GIS methods[29]. Our third measure was the average distance to the nearest park/green space. The Network Analyst extension of ArcGIS 9.2 was used to determine the distance from each child's residential postal code location to the nearest park/green space centroid. The average of these nearest distances was calculated for each community. Distance was calculated along the road network using the DMTI Spatial CanMap^® ^RouteLogistics file[41]. Our fourth measure of parks/green space access was the proportion of park/green space service area as a proportion of the total area within a community. Network Analyst was used to create 800 meter service areas around each park/green space based on the CanMap^® ^RouteLogistics file[41]. This distance has been specified as the maximum distance residents will walk to reach a community park/green space in a Canadian city with similar geographic characteristics to Calgary[29]. This index gives us insight into the amount of area serviced by a particular park/green space, while taking into account the crossing of boundaries.

### Analytic Procedure

We applied multivariate multilevel regression methods to quantify the association between each of the four parks measures (second or community-level) and childhood overweight/obesity (first or individual-level), adjusting for first- and second- level covariates. Multilevel analytic methods can account for the clustering of children's observations within communities and allow for estimation of the effects of both individual-level and community-level variables[7,42]. We combined the overweight and obese categories due to the small number of children classified as obese (4.5%). Our model was a two-level, random-intercept logistic regression with childhood overweight/obesity as the binary outcome (1 = overweight/obese; 0 = normal or underweight). Sex and dissemination area median family income (proxy for individual family income) were considered as individual-level covariates. For our four measures of parks/green space, we treated the variable as continuous when the distribution appeared normal, and created tertiles when the distribution was markedly non-normal. Each park variable was examined separately. Community-level education and proportion of visible minorities were examined as community-level covariates.

## Results

The mean age of the children was 4.95 years (range 3.0 to 8.01 years). Although most children received this vaccination between the ages of 4 and 6 years, some children are immunized throughout the school year, and therefore may be slightly older[43]. The median DA income (proxy for individual family income) for Calgary was $70,390 (range $11,006 to $246, 151). The mean proportion of adults with at least a completed Bachelor's degree was 23.8% (2.7% to 67.3% range) and the mean proportion of visible minorities in the community was 18.3% (2.7% to 67.6% range).

From the total 8,401 children in the original study, the following exclusions were made: 427 due to failure to geocode (i.e., missing postal code), 676 due to an insufficient number of records within a geographic unit (i.e., data for fewer than 20 children within a community), 70 due to missing BMI information, 2 due to being an outlier for age (defined as plus or minus 3.29 for an age z-score [44]), and 454 due to lack of income information in outlying areas in 2001. The final sample contained 6,772 children (3,332 girls and 3,450 boys).

Of this sample, 16.1% of children were either overweight or obese (16.3% girls, 15.9% boys). We had information on 1,559 of the children removed from the analysis and these children were not significantly different from those included in regards to sex or overweight/obesity proportion. Within Calgary's boundary, there were 862 areas designated as 'public park, school or recreation' covering 79 km^2 ^or 10.9% of the city's total area. Once the larger parks/green spaces were removed, 852 parks remained for inclusion in the analysis covering 42 km^2^. Our four measures of parks/green space access had the following distributions: number of parks/green space per 10,000 residents (range 0 to 150.7), proportion of parks/green space area (range 0 to 23%), average network distance to a park/green space (256.2 to 4,639.2 m), and proportion of parks/green space service area (0 to 100%).

Beginning with 185 communities, we removed communities with data for fewer than 20 children [45] (n = 68) and communities with missing information for DA income (n = 12). The resulting 105 communities had an average area of 3.0 km^2 ^(range 0.8 to 6.8 km^2^), and a mean population of 6,811 people (range 430 to 17,075).

The random intercept model allowed us to first ascertain that there was significant between-community variation in the outcome variable (overweight/obesity); p = < .001. We then systematically entered individual and community-level variables. Table 1 shows both the partially adjusted (sex and DA income) and fully adjusted (sex, DA income, community-level education, and community % visible minority) models for each parks/green space variable. Across all four parks/green space variables, we observed only one marginal finding: a moderate number of parks/green space per 10,000 people at the community level was associated with reduced risk of childhood overweight/obesity compared to a low number of parks/green space per 10,000 people at the community level (p = 0.066). In the fully adjusted model the effect was reduced to non-significance, although the actual size of the effect was only slightly reduced (OR changed from 0.84 to 0.89). As seen in Table 1, the effects of our three other parks/green space variables (proportion of parks/green space area, average network distance to a park/green space, and proportion of parks/green space service area) were non-significant in both the partially and fully adjusted models. The analysis was repeated including parks/green space over one km^2 ^and the results did not change (data not shown).

**Table 1:** Partially and fully adjusted multilevel models examining the association between four different measures of spatial access to parks/green space and childhood overweight/obesity.

	Partially Adjusted (Sex and Income)		Fully Adjusted (Sex, Income, Education, Visible Minority)	
	Odds Ratio (95% CI)	P-value	Odds Ratio (95% CI)	P-value
**Park Variable (Parks/10,000)**				
Parks/10,000 (Low)	1		1	
Parks/10,000 (Moderate)	0.84 (0.69-1.01)	p = 0.066	0.89 (0.75-1.06)	p = 0.193
Parks/10,000 (High)	0.92 (0.75-1.15)	p = 0.463	1.02 (0.83-1.26)	p = 0.857
Sex (female)	1.02 (0.88-1.17)	p = 0.830	1.01 (0.88-1.17)	p = 0.856
Income (DA level)	0.10 (1.00-1.00)	p < 0.001	0.10 (1.00-1.00)	p = 0.000
Completed at least Bachelor's degree (%)	-	-	0.26 (0.12-0.55)	p = 0.001
Visible Minority (%)	-	-	1.43 (0.78-2.62)	p = 0.246
				
**Park Variable (Proportion of Park Area)**				
Proportion of Park Area	0.54 (0.07-3.95)	p = 0.537	0.55 (0.08-3.90)	p = 0.550
Sex (female)	1.01 (0.88-1.17)	p = 0.839	1.01 (0.88-1.17)	p = 0.864
Income (DA level)	0.10 (1.00-1.00)	p < 0.001	0.10 (1.00-1.00)	p = 0.000
Completed at least Bachelor's degree (%)	-	-	0.27 (0.12-0.57)	p = 0.001
Visible Minority (%)	-	-	1.49 (0.81-2.70)	p = 0.199
				
**Park Variable (Average Network Distance)**				
Average Network Distance to Park (Low)	1		1	
Average Network Distance to Park (Moderate)	0.94 (0.77-1.16)	p = 0.564	0.88 (0.72-1.07)	p = 0.190
Average Network Distance to Park (High)	0.88 (0.72-1.07)	p = 0.201	0.87 (0.73-1.04)	p = 0.127
Sex (female)	1.01 (0.88-1.17)	p = 0.858	1.01 (0.88-1.17)	p = 0.900
Income (DA level)	0.10 (1.00-1.00)	p < 0.001	0.10 (1.00-1.00)	p = 0.001
Completed at least Bachelor's degree (%)	-	-	0.25 (0.11-0.55)	p = 0.001
Visible Minority (%)	-	-	1.50 (0.83-2.73)	p = 0.183
				
**Park Variable (Proportion of Park Service Area)**				
Proportion of Park Service Area	1.35 (0.82-2.22)	p = 0.235	1.35 (0.83-2.18)	p = 0.225
Sex (female)	1.01 (0.88-1.17)	p = 0.855	1.01 (0.88-1.16)	p = 0.879
Income (DA level)	0.10 (1.00-1.00)	p < 0.001	0.10 (1.00-1.00)	p = 0.001
Completed at least Bachelor's degree (%)	-	-	0.27 (0.13-0.56)	p = 0.001
Visible Minority (%)	-	-	1.51 (0.84-2.72)	p = 0.169

For all models, examining the individual-level variables revealed that increasing income (DA level) was associated with reduced risk of overweight/obesity though the effect was very small. Sex had no significant effect. Examining community-level variables revealed that increasing community-level education was associated with a reduced risk of childhood overweight/obesity, while community proportion of visible minorities had no significant effect.

**Figure 1:**
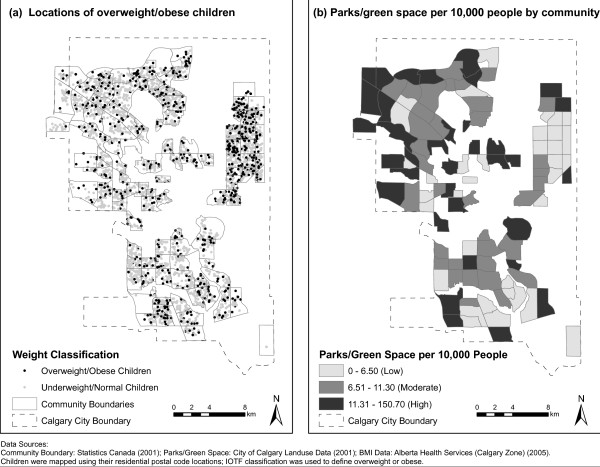
**Childhood Overweight/Obesity, and Parks/Green space per 10,000 in Calgary communities**.

**Figure 2:**
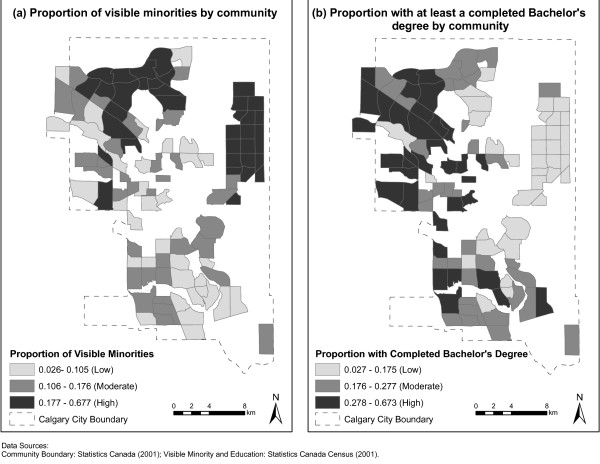
**Proportion of people with at least a completed Bachelor's degree and Proportion of Visible Minorities in Calgary Communities**.

Figure 1 illustrates the patterns for a) overweight/obesity across Calgary and b) number of parks/green space per 10,000 people by community. There is a clear pattern whereby communities that have more overweight/obese children tend to be in the lowest tertile for number of parks/green space per 10,000 people. Figure 2 illustrates a) proportion of visible minority by community and b) proportion with at least a completed Bachelor's degree by community. Figure 2 illustrates that the marginal finding for having a moderate versus low number of parks/green space per 10,000 people, in the partially adjusted model is a result of the patterns for community-level education and proportion of visible minority.

To explore the possibility that the influence of parks/green space variables differed by sex, or by income, we conducted post hoc testing of each parks/green space variable as a predictor of the sex-overweight/obesity association, and the income-overweight/obesity association. In no instance were significant interactions detected (data not shown).

In fully adjusted models, the variance component of the intercept remained significant (p-values ranged from 0.026 to 0.034), indicating that between-community variation in children's overweight/obesity remains and reflects unmeasured variables above and beyond those included here.

## Discussion

We examined the associations between four measures of spatial access to parks/green space at the community level and childhood overweight/obesity in Calgary, Canada. Overall, our results suggest that spatial access to parks/green space per se has a limited direct association with childhood overweight/obesity in this context. Nonetheless, our findings raise some interesting discussion points.

First, we observed marginal significance in the partially adjusted model where children living in communities with a moderate number of parks/green space per 10,000 people had lower odds of being overweight/obese compared to those living in communities with a low number of parks/green space per 10,000 people. In the fully adjusted model the effect was slightly reduced and became non-significant. Although our finding for number of parks/green space per 10,000 was small and non-significant we feel that this finding merits a brief comment given the local circumstances in Calgary. Currently, the City of Calgary's planning guidelines advocate the development of one centrally located park within new communities as opposed to several small parks[46]. Given the possibility that the number of parks/green space in and around a child's community may be associated with childhood overweight/obesity our results raise the suggestion that City guidelines should be periodically reconsidered.

However, we have to keep in mind that the effect of the number of parks/green space per 10,000 residents was not significant once we controlled for community-level education and proportion of visible minorities, speaking to the importance of these sociodemographic circumstances in relation to obesity as identified in other studies[47,48]. For example, Janssen and colleagues (2006) found that children living in communities with a high percentage of residents with less than a high school education had an increased likelihood of both obesity and unhealthy eating[47]. Since education can be used as a proxy for health literacy [49] it may represent a community's collective set of attitudes and beliefs about obesity including social norms related to diet and/or exercise in a community[50]. Review articles have discussed how U.S. communities with a high proportion of minorities have reduced neighbourhood access to supermarkets that provide access to a mix of healthful food products at reasonable cost and higher than average exposure to fast food restaurants [51,52] all of which may contribute to childhood obesity. These findings suggest that both ethnic and education factors at the community level (including culture, norms and health knowledge, beliefs, values and behaviours) are likely important factors in determining obesity risk.

Examining our individual-level variables in all of our models, we observed that DA income (as a proxy for individual family income) was negatively associated with overweight/obesity though the effect was small. This finding is consistent with Canadian studies that have shown an inverse association between various measures of family and/or household income (e.g., parent reported household income, youth perceived family wealth) and childhood obesity risk [47,53,54]. Recent studies provide several plausible explanations for the association between family and/or household income and childhood obesity. First, children from higher income families are more likely to have a family structure including two parents [55] who are more involved in their physical activities and also have the time to frequent other activities with them[56]. Additionally, two-parent families typically include a father in the home, and a recent review highlighted that fathers have been shown to be the most important role model for physical activity in young children[25]. Children from families with a higher income may also be more likely to have access to other important determinants of body weight including healthy foods [27,57] and have more opportunity to participate in organized sports and other physical activity pursuits that are often costly and require parental support[58,59]. Overall, our finding that higher family income (even when using DA level income as a proxy) is associated with reduced risk of overweight/obesity in children is consistent with the above Canadian studies.

Our other measures of parks/green space access, proportion parks/green space area, average network distance to the nearest park/green space, and proportion of parks/green space service area were not associated with childhood overweight/obesity. Our null findings on these variables differed from findings of two recent studies that have reported that greater neighbourhood park access was associated with lower risk of overweight/obesity[26,27]. These inconsistent findings may reflect differences in our methodologies with one of the above studies examining access to a variety of physical activity facilities combined with parks and obesity in grade 7 to 12 students [26] and the other examining parent's subjective measures of park access and overweight/obesity in grade 5 students [27]. Methodological considerations may also explain our null finding for proportion of parks/green space service area. While the use of this particular variable is common in the spatial accessibility literature [29] there is an issue with this measure in the context of Calgary. Parks/green space service area does not account for community size and bases its measure on number of parks/green space as opposed to size of the parks/green space. Therefore, this measure may not be appropriate for use in cities, like Calgary, where community size varies drastically. We also found no significant association for proportion of parks/green space but note that this may have resulted from the small range and thereby relatively equitable distribution of proportion of parks/green space across Calgary communities.

Our null finding for average distance to a park/green space is consistent with another study that found no association between average distance by street travel to the nearest park and childhood obesity in a population of US low-income pre-schoolers[28]. Additionally, this null finding supports the idea that average distance to parks/green space may not be an important feature of the built environment in communities like Calgary that are characterized by high car use. A recent Canadian study showed that, while 49% of parents reported frequenting the park closest to their home, the majority of respondents reported travelling more than 4 km to get to their desired park[60]. For those parents who chose to travel to a park, park location was not as important as park amenities[60]. Given that Calgary is a city characterized by a high amount of car travel, it seems plausible that whether parks are nearby or not is perhaps irrelevant to many Calgarians, who would opt to drive to a more desired park [32,33] or to drive their child to organized activities.

Considering the consistency of our null findings across all of the fully adjusted models, it is possible that spatial access to parks/green space is truly not pertinent to overweight/obesity in young children living in cities similar to Calgary. This conclusion is supported by another Canadian study, conducted in a city comparable to Calgary, that found no association between the number of physical activity facilities, including parks and playgrounds, and childhood overweight/obesity in a similar population of young Canadian children[23]. It is also possible that our findings reflect a disconnect between physical activity and obesity; namely, perhaps our measures of parks/green space access has implications for physical activity in Calgary (which we did not measure), but that its implications do not show up when we examine overweight/obesity. This would be an important question for future research. Also important is the fact that access to parks/green space does not equal utilization and therefore gaps remain in terms of clarifying the associations between access to parks/green space, actual usage, and the implications - if any - for weight. It is also possible that Calgary's climate, characterized by long and sometimes very cold winters precludes parks/green space, despite their location, from being an important resource for children.

One limitation of our study is the temporal disconnect between census data (from 2001, the most current available at time of writing) and the height and weight data which were collected in 2005. As a result, some children's postal codes corresponded to a community that had not been developed at the time of the 2001 census. These children had to be excluded from the analysis, which led to a reduction in the effective sample size both for children and for communities. Another limitation is that not all families choose to vaccinate their children and it is possible that these families are of lower socioeconomic status[43]. Since there is evidence to suggest that children of lower SES may be at increased risk of overweight/obesity [47,53,61] this study may underestimate the prevalence of overweight/obesity and may be underestimating the effects of parks/green space access on childhood overweight/obesity. And finally, the cross-sectional nature of the data clearly precludes discussions about causality. Strengths of this study include the high quality of the height and weight data, which were obtained from directly measuring a large number of Calgary children reporting to vaccination clinics. Also, the use of GIS allowed for full coverage of Calgary and objectively measured data for each parks/green space variable. Furthermore, this study's examination of four different measures of spatial access to parks/green space allowed us to tap into the breadth of ways to measure parks/green space access.

There is an important opportunity to further this line of research by examining ongoing development of new communities in relation to the overweight/obesity status of children. This is especially relevant in the Calgary context, since the city is experiencing tremendous growth with many new community designs being constructed. Thus, Calgary's unique context provides an important opportunity for future research to examine several characteristics of the built environment, including the effects of current park/green space planning policies[46]. Since the development of childhood overweight/obesity is complex and multifactorial, future research studies need to examine a more exhaustive set of possible etiological factors. Overall, the findings of this research suggest that the relevance of spatial access to parks/green space in terms of its association with childhood overweight/obesity are likely context specific and in some cities such as Calgary, having good spatial access to parks/green space may not be an important feature of the built environment.

## Conclusion

This study's results indicate that the association between spatial access to parks/green space and childhood overweight/obesity may be different in various urban contexts. Our null findings in Calgary, Canada suggest that spatial access to parks/green as a feature of the built environment may be less relevant to childhood overweight/obesity in car dependent cities and communities.

## Competing interests

The authors declare that they have no competing interests.

## Authors' contributions

MP contributed to the conceptualization the study and the statistical analysis, led the interpretation of the results, and drafted the manuscript. AP contributed to the statistical analysis and interpretation of results. CP contributed to data management and statistical analysis. DM contributed to the design of the study and acquisition of the data. DJ contributed to the conceptualization of the study and the interpretation of results. LM contributed to the conceptualization of the study and supervised the interpretation of the results and the writing of the manuscript. All authors have read and approved the final manuscript.
